# Short-Term Simulated NH_4_^+^/NO_3_^−^ Inputs Reorganize Soil Microbial Communities and Modify N-Cycling Functional Potential in Temperate Forests

**DOI:** 10.3390/microorganisms14102323

**Published:** 2026-10-12

**Authors:** Yunzhe Hu, Shulan Cheng, Huajun Fang, Mingzhu Lu

**Affiliations:** 1 College of Resources and Environment, University of Chinese Academy of Sciences, Beijing 100049, China; huyunzhe25@mails.ucas.ac.cn; 2 Key Laboratory of Ecosystem Network Observation and Modeling, Institute of Geographic Sciences and Natural Resources Research, Chinese Academy of Sciences, Beijing 100101, China; 3 The Zhongke-Ji’an Institute for Eco-Environmental Sciences, Ji’an 343000, China; 4 Hubei Key Laboratory of Wetland Evolution & Ecological Restoration, Wuhan Botanical Garden, Chinese Academy of Sciences, Wuhan 430074, China; lumingzhu@wbgcas.cn

**Keywords:** nitrogen deposition, soil N_2_O emissions, NH_4_^+^/NO_3_^−^ ratio, soil microbial community, metagenomics

## Abstract

Forest soil nitrogen (N) turnover and nitrous oxide (N_2_O) release are strongly influenced by atmospheric N deposition. Although the effects of individual inorganic N species on soil biogeochemistry are well documented, the consequences of changing their relative supply for N_2_O formation and the underlying microbial pathways remain less clear. We therefore examined soil N_2_O emissions, microbial communities, and N-transformation potential across a gradient of ammonium-to-nitrate (NH_4_^+^/NO_3_^−^) inputs in a temperate forest soil from Northeast China. Seven treatments covering NH_4_^+^/NO_3_^−^ ratios from 1:5 to 5:1 were established. Soil physicochemical properties and N_2_O emissions were measured, while microbial taxonomic profiles and N-cycling genes were characterized by metagenomic sequencing. Altering the composition of inorganic N reorganized the microbial community, including the relative representation of archaeal, bacterial, and eukaryotic groups. N_2_O release was greatest when NH_4_^+^ and NO_3_^−^ were supplied in equal proportions, a pattern that may reflect a stronger contribution of nitrification-associated processes. Across the treatment gradient, the response of N_2_O emissions to inorganic N composition was nonlinear. By contrast, the 1:2.5 NH_4_^+^/NO_3_^−^ treatment produced comparatively low N_2_O emissions and contained a greater representation of genes associated with N_2_O reduction, consistent with enhanced microbial potential for N_2_O consumption. Under the more nitrate-dominated 1:5 treatment, genes involved in dissimilatory nitrate reduction to ammonium (DNRA) were enriched, indicating a shift in the potential routes of nitrate transformation. Metagenomic data further revealed marked variation in genes linked to nitrification, denitrification, and biological N fixation. In comparison with these compositional and functional responses, microbial diversity changed relatively little, possibly because overlapping functions were distributed among different taxa under contrasting N inputs. Overall, the findings show that the balance between ammonium and nitrate can affect forest-soil N_2_O emissions by restructuring microbial assemblages and their potential to transform N. These responses emphasize the need to consider the composition, rather than only the amount, of N deposition when evaluating microbial regulation of ecosystem N cycling and greenhouse-gas release. Field studies are still needed to test the generality of these patterns and their relevance to N management.

## 1. Introduction

Since industrialization, human activities have accelerated the transfer of reactive nitrogen (N) from the atmosphere to terrestrial ecosystems. Fossil-fuel combustion, application of synthetic N fertilizers, and livestock production are major contributors to the worldwide increase in atmospheric N deposition [1]. Current estimates place global mean deposition above 10 kg N ha^−1^ yr^−1^, and one projection suggests that it may approach 50 kg N ha^−1^ yr^−1^ by 2050 [2]. China has experienced particularly high deposition relative to many other regions: the reported annual rate increased from 13.2 kg N ha^−1^ yr^−1^ in 1980 to 21.2 kg N ha^−1^ yr^−1^ in 2000 [3], and the national mean during 2000–2015 was 20.4 ± 2.6 kg N ha^−1^ yr^−1^ [4]. Changes have occurred not only in deposition magnitude but also in the composition of inorganic N. In northeastern China, a reported time series showed a decline in the ammonium-to-nitrate (NH_4_^+^/NO_3_^−^) ratio from 2.47 to 1.17 over roughly two decades [4], coincident with shifts in energy consumption and emission-control policies. Ecological outcomes depend partly on the amount of N supplied. Moderate deposition can alleviate N limitation, enhance plant growth, and promote carbon storage [5,6,7], whereas chronic excess may acidify soil, alter microbial diversity, and increase nitrous oxide (N_2_O) emissions, thereby impairing forest ecosystem functioning [8,9].

Additional N supply can change both N-transformation pathways and greenhouse-gas fluxes in temperate forest soils. Greater mineral N availability may accelerate mineralization, nitrification, and denitrification, thereby affecting the formation of N_2_O [10,11,12]. In some middle- and high-latitude temperate forests, N enrichment has increased N_2_O emissions to two or three times the levels measured in untreated soils [8]. The chemical form of added N is also important because it determines which microbial processes receive substrate. Ammonium (NH_4_^+^), for example, can support ammonia oxidation by organisms including ammonia-oxidizing bacteria (AOB), whereas nitrate (NO_3_^−^) can promote heterotrophic denitrification when environmental conditions are favorable [13,14]. Over longer periods, N loading may also shift soil pH and select nutrient-responsive microorganisms at the expense of oligotrophic taxa, with consequences for the communities responsible for N_2_O production and consumption [15]. However, much of the existing evidence is based on additions of only one inorganic N form. Mixed N inputs, variation in NH_4_^+^/NO_3_^−^ proportions, and the contribution of organic N have been examined less systematically [16,17]. As a result, the microbial processes linking changes in deposited-N composition to soil N_2_O emissions remain incompletely understood.

The effects of changing N inputs are likely mediated by interactions between soil chemistry and microbial community structure. Enrichment with N can modify soil pH, C/N balance, and mineral N pools, thereby selecting different microbial taxa and metabolic functions [15,18,19,20]. Reported community responses include reduced bacterial diversity, enrichment of nutrient-responsive Proteobacteria and Bacteroidetes, and declines in oligotrophic Acidobacteria, whereas fungal diversity may remain relatively stable or increase slightly [20]. At the functional level, N addition can alter the abundance of genes involved in ammonia oxidation (*amoA*), nitrite reduction (*nirK* and *nirS*), and N_2_O reduction (*nosZ*), as well as the relative involvement of AOB and ammonia-oxidizing archaea (AOA) in nitrification [19]. Long-term enrichment has additionally been linked to simpler microbial co-occurrence networks and changes in the roles of highly connected taxa [21,22]. Nevertheless, experiments using a single N source or only a limited range of addition levels cannot adequately capture microbial responses to contrasting inorganic N mixtures [16]. Determining how taxonomic composition and N-cycling functional potential vary across NH_4_^+^/NO_3_^−^ ratios is therefore essential for evaluating the ecological consequences of changes in deposition composition.

Progress on this question has also been limited by methodological constraints. Amplicon sequencing of bacterial 16S rRNA genes or fungal internal transcribed spacer regions can resolve selected components of community composition, but it does not directly inventory the broader suite of genes involved in N metabolism [23]. Shotgun metagenomics, in contrast, permits microbial taxonomic composition and encoded functional capacity to be examined within the same dataset [24]. Previous studies have shown that prolonged fertilizer inputs can modify microbial metabolic potential [25,26], while soil N_2_O dynamics emerge from a network of interacting microbial transformations rather than a single process [27]. Alongside conventional autotrophic nitrification and heterotrophic denitrification, processes such as nitrifier denitrification, nitrite oxidation, anaerobic ammonium oxidation, and dissimilatory nitrate reduction to ammonium (DNRA) can change N pools and substrate availability and may thereby affect N_2_O production or consumption [27,28]. Metagenomic analysis can identify taxa and genes associated with these pathways and support mechanistic hypothesis development [29,30,31], although the presence or abundance of genes alone does not demonstrate pathway activity or quantify reaction rates.

Regional trends provide an additional reason to examine variation in N composition. One estimate for the region reported atmospheric N deposition values of 16.27, 22.71, and 21.02 kg N ha^−1^ yr^−1^ at successive time points [4]. Other estimates indicate that organic forms account for approximately 30% of deposited N and that the regional NH_4_^+^/NO_3_^−^ ratio declined from 4.82 to 1.39 between 1980 and 2015 [4,32]. Although nitrate and ammonium additions have previously been shown to produce different soil N_2_O responses [8,16], the effects of systematically changing their relative proportions are still poorly resolved. For instance, at an N loading of 80 kg N ha^−1^ yr^−1^, one study observed greater N_2_O release after nitrate addition than after ammonium addition [16]. To address this gap, we established seven NH_4_^+^/NO_3_^−^ treatments and coupled soil measurements with metagenomic sequencing to characterize N_2_O fluxes and microbial responses. We proposed that (1) the balance between inorganic N forms would modify the taxonomic composition and functional potential of archaeal, bacterial, and eukaryotic communities; (2) increasingly nitrate-dominated inputs would enhance N_2_O emissions and alter the relative contribution of microbial N-transformation pathways; and (3) DNRA, in addition to nitrification and denitrification, could contribute to the microbial N transformations associated with N_2_O release. The study was designed to clarify how shifts in atmospheric N deposition composition may influence N cycling and greenhouse-gas emissions in temperate forest soils.

## 2. Materials and Methods

### 2.1. Site Description and Soil Collection

Soils were collected from the national forest ecosystem observation and research station on Changbai Mountain in Jilin Province, China (42°24′ N, 128°6′ E; approximately 750 m a.s.l.). The region has a mountainous temperate continental climate, with a growing season from May to September. Mean annual temperature is 3.6 °C; mean temperatures in July and January are 19.7 °C and −12.5 °C, respectively. Mean annual precipitation is approximately 745 mm, and nearly 80% falls during the growing season [19]. The sampling location has a slope of <10°, a groundwater table at approximately 9 m depth, and its highest surface moisture generally occurs during spring snowmelt.

The sampling stand is a broad-leaved Korean pine forest approximately 200 years old and is representative of the temperate mixed coniferous–broadleaf forest zone. *Pinus koraiensis* occurs with four dominant broad-leaved tree species: *Acer mono*, *Fraxinus mandshurica*, *Quercus mongolica*, and *Tilia amuresis*. The soil, classified as mountain dark brown soil (Eutric Cambisol according to the FAO system), developed from volcanic-ash gravel parent material. It is well drained and has a depth of 70–100 cm. Previously reported baseline characteristics were a bulk density of 0.56 g cm^−3^, dissolved organic carbon (DOC) of 11.45%, a C/N ratio of 21.84, total carbon (TC) of 156.6 g kg^−1^, total nitrogen (TN) of 7.17 g kg^−1^, total phosphorus (TP) of 0.97 g kg^−1^, total potassium (TK) of 12.2 g kg^−1^, and a pH of 4.66.

Sampling was conducted in July 2019. After removing the surface litter, approximately 20 kg of soil was collected from the 0–20 cm depth at a relatively level and minimally disturbed location. Surface material originating from litter was defined as the organic layer, whereas the underlying mineral soil was considered the mineral layer. The collected material was composited, passed through a 2-mm sieve to improve homogeneity, and air-dried at room temperature for 15 days until moisture content was approximately 21.85%. After this pretreatment, NH_4_^+^-N and NO_3_^−^-N concentrations were 159.67 and 113.94 mg kg^−1^, respectively, and soil pH was 4.94 [19].

### 2.2. Experimental Design

The single-factor incubation experiment included eight groups: an N-free control (CK) and seven treatments with NH_4_^+^/NO_3_^−^ input ratios of 5:1, 2.5:1, 1.5:1, 1:1, 1:1.5, 1:2.5, and 1:5. Treatments with the first four ratios were labeled A5, A2.5, A1.5, and AN1, whereas the final three were labeled N1.5, N2.5, and N5. Each group contained four independent biological replicates, yielding 32 incubation vessels in total; individual bottles were treated as separate experimental units [16].

For every N-amended treatment, total N addition corresponded to 80 kg N ha^−1^ yr^−1^. Urea-N accounted for 30% of the applied N, while the remaining 70% was supplied as NH_4_Cl-N and NaNO_3_-N at proportions defined by the respective inorganic-N treatment. Consequently, the nominal NH_4_^+^/NO_3_^−^ ratios refer to the proportions of directly applied NH_4_^+^-N and NO_3_^−^-N within the inorganic N component and do not represent the actual NH_4_^+^/NO_3_^−^ ratio in the soil during incubation. Each 250-mL glass vessel received 115 g of air-dried soil, equivalent to approximately 90 g on a dry-soil basis, and retained a headspace of about 100 cm^3^. NaNO_3_, NH_4_Cl, and urea were dissolved in ultrapure water and distributed uniformly over the soil to establish the specified treatments. The same volume of N-free water was added to CK vessels.

Prior to N addition, the air-dried soil was rewetted and incubated in darkness for 7 days. This pre-incubation was used to allow microbial activity to recover from the immediate rewetting disturbance and to attenuate the temporary mineralization pulse produced by the dry–rewet transition. The designated NH_4_^+^/NO_3_^−^ treatments were then applied, soil moisture was adjusted to 60% water-filled pore space (WFPS), and an 84-day incubation was conducted at 20 °C. Water was added every two days to maintain moisture. At the end of day 84, soil was sampled for physicochemical analyses and shotgun metagenomic sequencing.

### 2.3. Measurement of N_2_O Emissions and Soil Characteristics

N_2_O emissions were monitored repeatedly during the 84-day incubation, with more frequent observations early in the experiment to resolve short-term changes. Sampling was conducted on days 0, 1, 2, 3, 5, 7, 10, 12, 14, 17, 20, 23, 27, 31, 40, 46, 50, 56, 69, 75, and 84.

At each gas-sampling time, incubation vessels were sealed with rubber stoppers fitted with a needle and three-way valve. At 0, 15, 30, and 45 min after closure, 10-mL samples of headspace gas were withdrawn and transferred into evacuated vials. N_2_O concentrations in the gas phase were quantified with an Agilent 7890A gas chromatograph (Agilent, Santa Clara, CA, USA) equipped with an autosampler. Instantaneous N_2_O fluxes (μg N_2_O-N kg^−1^ dry soil h^−1^) were derived from the slope of the linear regression between headspace concentration and closure time. Cumulative N_2_O emissions were obtained by integrating the measured fluxes over the incubation period.

At the completion of the incubation, soil was analyzed for NH_4_^+^-N, NO_3_^−^-N, total dissolved nitrogen (TDN), DOC, dissolved organic nitrogen (DON), moisture content, and pH. NH_4_^+^-N, NO_3_^−^-N, and TDN were extracted with 2 M KCl and measured on an AA3 flow analyzer (SEAL, Norderstedt, Germany). DON was calculated as TDN minus the combined concentrations of NH_4_^+^-N and NO_3_^−^-N [33].

For DOC analysis, 5 g of organic-layer soil or 15 g of mineral-layer soil was mixed with 100 mL deionized water, shaken for 1 h, centrifuged for 30 min, and filtered through a 0.45-μm membrane. Soil pH was measured at a soil-to-water ratio of 1:2.5. Gravimetric water content was determined after oven drying at 105 °C for 24 h.

### 2.4. Metagenomic Sequencing and Bioinformatic Analysis

Immediately after the incubation ended, DNA was extracted from the soil. Four independent biological replicates from each treatment were analyzed by metagenomic sequencing. DNA concentration and purity were evaluated with a PicoGreen fluorescence assay, and integrity was checked by agarose gel electrophoresis [33,34]. Samples that passed quality control were used to prepare paired-end whole-genome shotgun libraries. Sequencing was performed on an Illumina HiSeq platform using 150-bp paired-end reads and generated approximately 10 Gb of raw sequence data per sample.

Adapter sequences and low-quality reads were removed before downstream processing. Cutadapt v1.17 was used for adapter trimming with a minimum 3-bp overlap and a maximum mismatch proportion of 20%. The trimmed reads were then filtered with fastp v0.20.0 using a five-base sliding window. Reads were excluded if their mean quality was below Q20, their length was <50 bp, or they contained ambiguous bases. Only the resulting high-quality reads were retained for assembly and annotation.

Each sample was assembled independently with MEGAHIT v1.2.9 using default settings. Open reading frames were predicted from the assembled contigs with Prodigal v2.6.3 in metagenomic mode. To generate a non-redundant gene catalog, predicted genes were clustered with CD-HIT v4.8.1 at 95% sequence identity and with alignment coverage of at least 90% of the shorter sequence. One representative sequence from each cluster was retained for subsequent analyses.

Taxonomic assignments were generated with DIAMOND v0.8.22 against the NCBI NR database and summarized at the phylum level. The non-redundant gene catalog was functionally annotated against the Kyoto Encyclopedia of Genes and Genomes (KEGG) database using DIAMOND BLASTP. Hits were accepted at an E-value ≤ 1 × 10^−5^, sequence identity ≥ 30%, and alignment coverage ≥ 70%. N-cycling genes were then identified from their KEGG Orthology assignments and classified according to the N-transformation pathways summarized in Table S1.

Gene abundance was quantified by mapping quality-filtered reads from each sample to the non-redundant gene catalog with Bowtie2 v2.5.2 using the ‘--sensitive’ (or ‘--very-sensitive’) and ‘--end-to-end’ options. Read counts were converted to transcripts per million (TPM), thereby normalizing for variation in gene length and sequencing depth among samples. The resulting normalized abundance profiles formed the basis for treatment comparisons of overall microbial functional composition and N-cycling genes.

Raw metagenomic reads together with the associated sample metadata are available in the NCBI Sequence Read Archive under BioProject/accession PRJNA717929.

### 2.5. Statistical Analyses

Data normality was assessed using the Shapiro–Wilk test. Variables that did not conform to a normal distribution were natural-log transformed before statistical analysis. One-way analysis of variance (ANOVA) was used to test treatment effects on N_2_O emissions, soil chemical properties, N-transformation gene abundances, and microbial community composition and diversity. When ANOVA indicated a significant treatment effect, Duncan’s multiple range test was applied at *p* < 0.05. Effect sizes were reported as η^2^.

Differences in microbial community composition among treatments were visualized by principal coordinates analysis (PCoA) using Bray–Curtis dissimilarities and tested statistically with permutational multivariate analysis of variance (PERMANOVA). Homogeneity of multivariate dispersion was checked with PERMDISP. For functional genes, each N-amended treatment was compared with CK. The Benjamini–Hochberg procedure was used to control the false discovery rate (FDR), and conclusions from these comparisons were based on FDR-adjusted *p* values; the corresponding effect sizes were also calculated. All statistical analyses and graphics were completed in R version 4.3.1 [35] with the vegan, ggplot2, and plyr packages.

## 3. Results

### 3.1. Soil N_2_O Emissions

**Figure 1:**
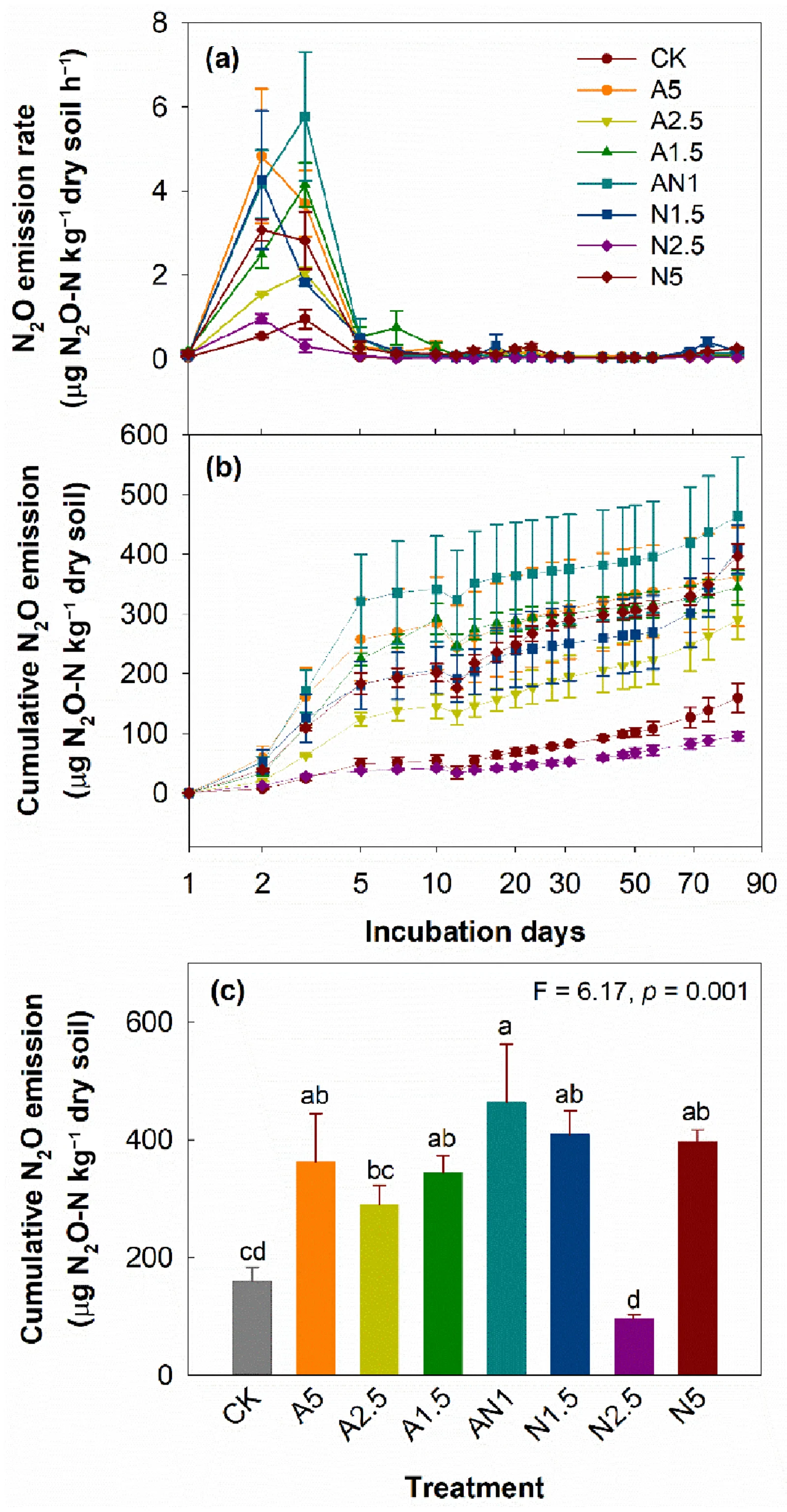
Temporal changes in N_2_O fluxes (**a**) and cumulative N_2_O release (**b**,**c**) over the 84-day incubation. CK is the control without N addition; A and N denote NH_4_^+^-N and NO_3_^−^-N additions, respectively, and treatment numbers indicate NH_4_^+^/NO_3_^−^ ratios. Values are means ± standard error. Different lowercase letters denote significant treatment differences in cumulative N_2_O emissions (*p* < 0.05).

N_2_O emissions were concentrated in the early phase of the 84-day incubation. Fluxes were greatest during the first 10 days, with peaks on days 2 and 3, and then declined rapidly before stabilizing (Figure 1a). During the first three days, AN1 generated the largest N_2_O flux, reaching 5.77 μg N_2_O-N kg^−1^ dry soil h^−1^ on day 3. The next highest values occurred in A5 and A2.5, at 3.69 and 2.04 μg N_2_O-N kg^−1^ dry soil h^−1^, respectively. Among N-amended treatments, N2.5 showed the smallest maximum flux (0.95 μg N_2_O-N kg^−1^ dry soil h^−1^), whereas CK had the lowest value overall (0.54 μg N_2_O-N kg^−1^ dry soil h^−1^). Fluxes declined in every treatment from day 3 to day 7, and the reduction was most pronounced in AN1. With the exception of A1.5, the N-enriched treatments subsequently fell to levels comparable with CK, with maximum rates no greater than 0.18 μg N_2_O-N kg^−1^ dry soil h^−1^ (Figure 1a). By the end of the experiment, cumulative N_2_O emissions followed the order AN1 > N1.5 > other N-enriched treatments > CK > N2.5 (Figure 1b,c). Thus, AN1 produced the strongest cumulative N_2_O response among the treatments and provides a reference point for evaluating N-input strategies.

### 3.2. Soil Physicochemical Properties

After 84 days, treatments did not significantly alter soil pH, NO_3_^−^-N, DOC, TC, TN, or the C/N ratio; NH_4_^+^-N was the only measured property showing a significant treatment effect (Table 1). Every N-amended treatment had a significantly higher NH_4_^+^-N concentration than CK, whereas NH_4_^+^-N did not differ significantly among the N treatments themselves. Treatments with NH_4_^+^/NO_3_^−^ ratios > 1 tended to have slightly greater NH_4_^+^-N, while the 1:1 treatment showed somewhat higher TC and TN than the other treatments (Table 1).

### 3.3. Composition and Diversity of Soil Microbial Communities

Across all treatments, metagenomic sequencing detected 3 domains (Archaea, Bacteria, and Eukaryotes), 185 phyla, 215 classes, 585 orders, 1305 families, 4598 genera, and 33,948 species. Bacterial reads were most numerous, eukaryotic reads ranked second, and archaeal reads were least abundant (Table 2). AN1 contained the largest read counts for all three domains. By contrast, N2.5 had the smallest archaeal and eukaryotic read counts, while N1.5 had the smallest bacterial read count (Table 2). In general, bacterial read counts were higher in treatments with NH_4_^+^/NO_3_^−^ ratios > 1 than in treatments with ratios < 1 (Table 2).

**Figure 2:**
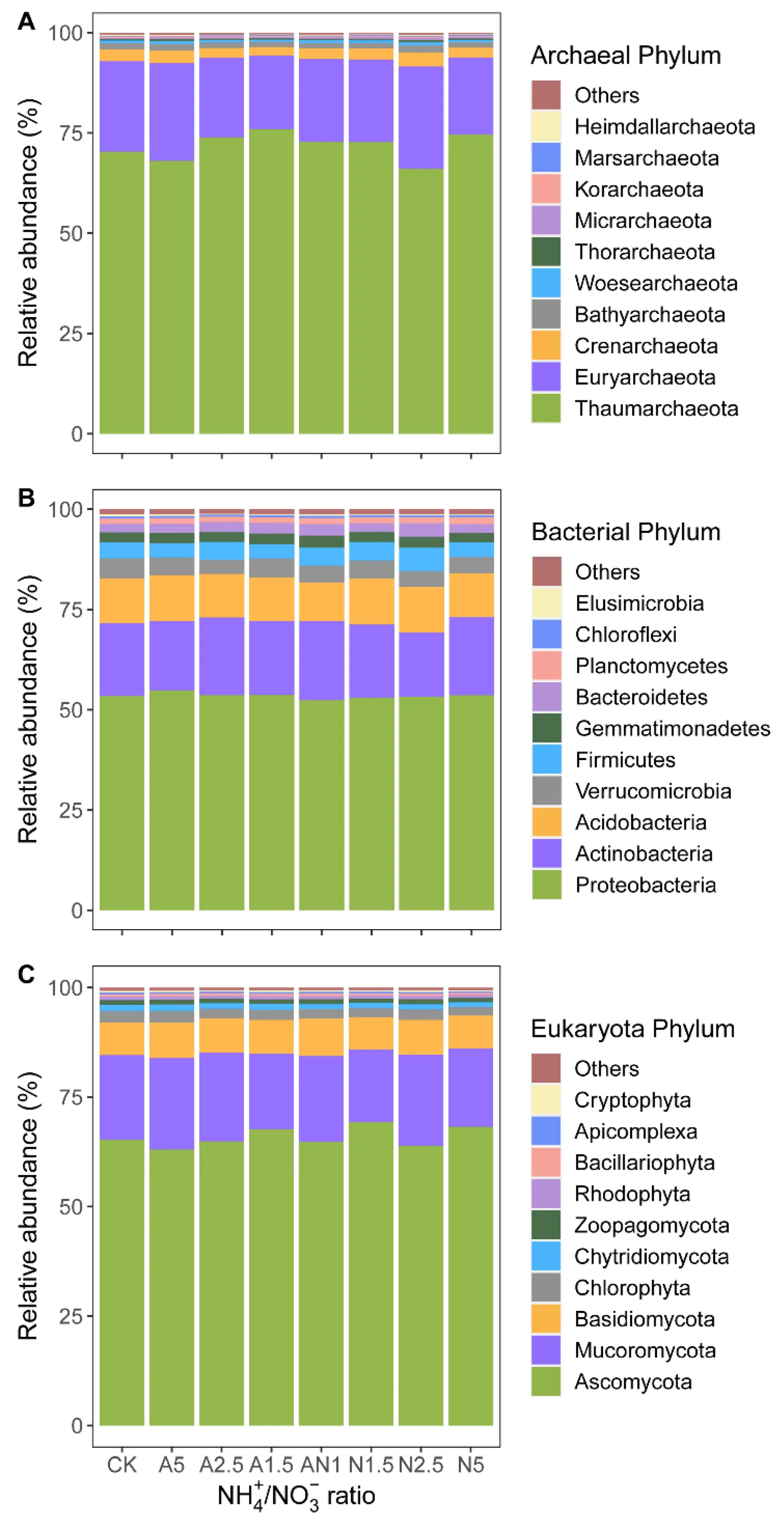
Relative abundances of the ten most abundant archaeal (**A**), bacterial (**B**), and eukaryotic (**C**) phyla across NH_4_^+^/NO_3_^−^ input treatments. CK is the N-free control; A and N indicate NH_4_^+^-N and NO_3_^−^-N additions, respectively, and the treatment numbers specify the NH_4_^+^/NO_3_^−^ ratios.

Thaumarchaeota dominated the archaeal assemblage (>65%), followed by Euryarchaeota (21.37%) and Crenarchaeota (2.73%). Relative to CK, Euryarchaeota was generally more abundant after N addition except in A5 and N2.5, and its maximum abundance (75.97%) occurred in A1.5. Thaumarchaeota tended to increase as the NH_4_^+^/NO_3_^−^ ratio declined. Among treatments with ratios < 1, its lowest abundance occurred in N2.5, and Thaumarchaeota was significantly negatively correlated with Euryarchaeota and Crenarchaeota (Figure 2A). Proteobacteria was the dominant bacterial phylum (53.48%), followed by Actinobacteria (18.32%). Within the N-enriched treatments, Proteobacteria ranged from 52.37% in AN1 to 54.80% in A5. Treatments with NH_4_^+^/NO_3_^−^ ratios < 1 generally contained more Proteobacteria than those with ratios > 1, and its relative abundance increased as the ratio declined. Acidobacteria reached its minimum in AN1 (9.62%) and remained near 11% in the other treatments (Figure 2B). Eukaryotic communities were dominated by Ascomycota (65.86%), with Mucoromycota accounting for 19.08%. Ascomycota ranged from 63.01% in A5 to 69.33% in N1.5. When the NH_4_^+^/NO_3_^−^ ratio was >1, Ascomycota became more abundant as the ratio decreased; at ratios < 1, abundance was higher in N1.5 and N5 than in N2.5 (Figure 2C).

**Figure 3:**
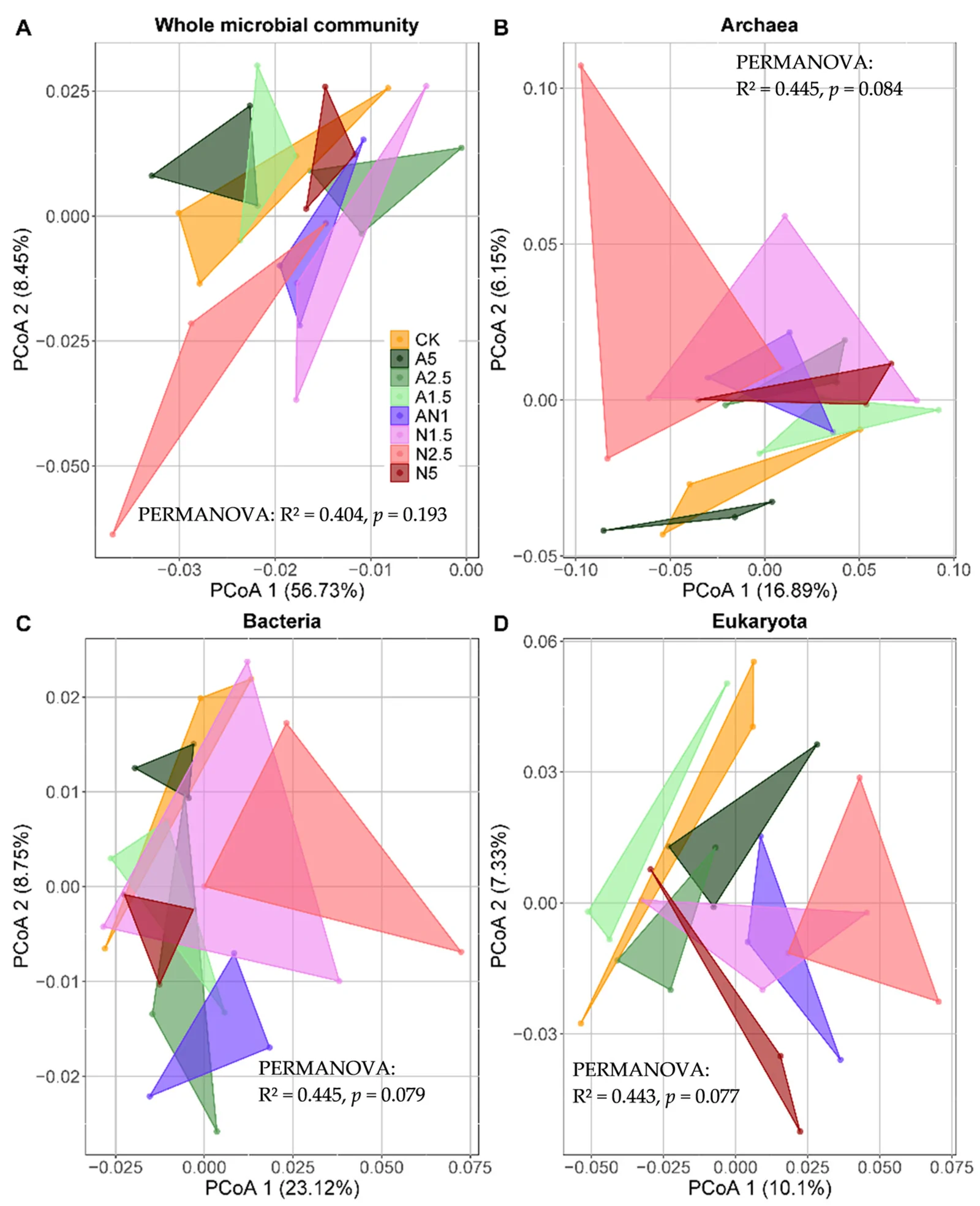
Principal coordinates analysis (PCoA) of the whole microbial community (**A**) and the archaeal (**B**), bacterial (**C**), and eukaryotic (**D**) communities at the phylum level. Treatment effects on community composition were tested by permutational multivariate analysis of variance (PERMANOVA) using community-distance matrices. CK denotes the control without N addition; A and N denote NH_4_^+^-N and NO_3_^−^-N additions, respectively, and the treatment numbers indicate NH_4_^+^/NO_3_^−^ ratios.

Neither Shannon diversity nor Sobs differed significantly among the NH_4_^+^/NO_3_^−^ treatments (Table 3). Nonetheless, archaeal Shannon diversity showed a slight increase under N enrichment, and Sobs values for archaea, bacteria, and eukaryotes were generally higher when the NH_4_^+^/NO_3_^−^ ratio exceeded 1 than when it was below 1 (Table 3). In the PCoA, communities from A5, A1.5, and N5 were positioned similarly to CK, whereas A2.5, AN1, N1.5, and N2.5 showed significant differences, especially under NH_4_^+^/NO_3_^−^ ratios < 1 (Figure 3). PERMDISP detected no significant among-treatment differences in within-group multivariate dispersion (*p* > 0.05; Figure 3).

**Figure 4:**
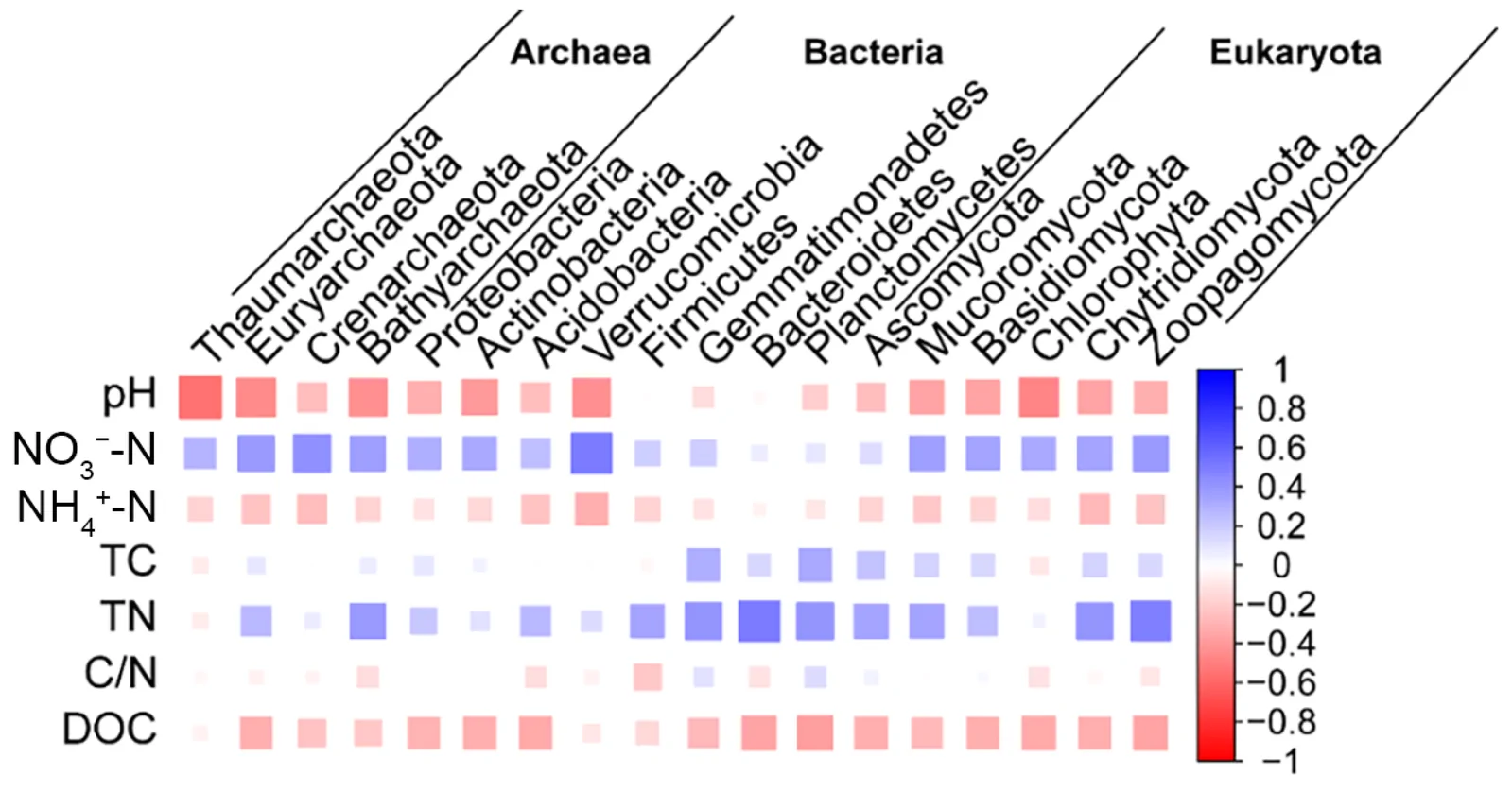
Pearson-correlation heatmap relating soil biochemical properties to the abundances of dominant archaeal, bacterial, and eukaryotic phyla. Blue and red cells denote positive and negative correlations, respectively. Cell size and color intensity reflect correlation strength, with darker shading indicating stronger relationships. Filled squares mark significant correlations (*p* < 0.05); blank positions indicate nonsignificant correlations.

With the exception of Firmicutes and Bacteroidetes, most dominant phyla were significantly negatively associated with soil pH, positively associated with NO_3_^−^-N, and negatively associated with NH_4_^+^-N. Dominant bacterial groups were also positively related to TN and negatively related to DOC (Figure 4). These relationships indicate that microbial community composition responded strongly to N-source conditions and point to an important association between the NH_4_^+^/NO_3_^−^ balance and microbial community diversity and structure.

### 3.4. Changes in Functional Genes Related to Soil N Metabolism

**Figure 5:**
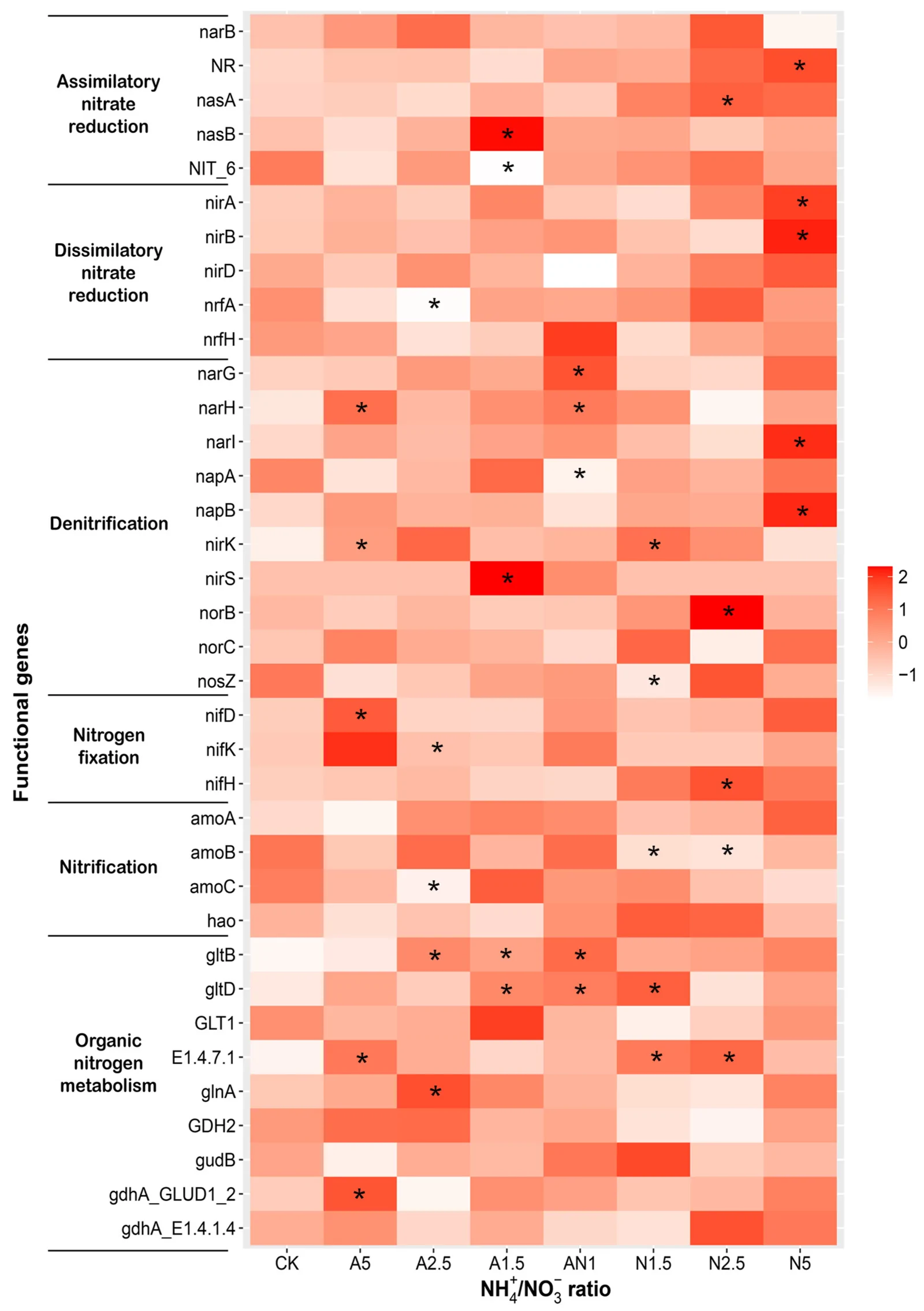
Heatmap showing relative abundances of N-metabolism genes across NH_4_^+^/NO_3_^−^ treatments. An asterisk (*) denotes a Benjamini–Hochberg FDR-adjusted *p* value < 0.05. CK is the untreated control; A and N indicate NH_4_^+^-N and NO_3_^−^-N additions, respectively, and the treatment numbers denote NH_4_^+^/NO_3_^−^ ratios.

Annotation against KEGG identified 36 genes associated with N metabolism. These genes were assigned to seven functional categories: nitrification, denitrification, N fixation, nitrate assimilation and reduction (ANRA), DNRA, anammox, and organic N metabolism. No anammox-associated genes were detected in any treatment, indicating that the corresponding functional potential was either low or below the detection limit of the metagenomic dataset under the present conditions (Figure 5). Overall, treatments with NH_4_^+^/NO_3_^−^ ratios > 1 contained higher abundances of genes associated with denitrification, ANRA, and DNRA, with especially pronounced values in N5 (Figure 5). Genes involved in N fixation were more abundant in A5, whereas nitrification-related genes were more abundant in A2.5 and A1.5 (Figure 5). Thus, the relative abundance of N-metabolism genes varied significantly with the composition of the N input.

**Figure 6:**
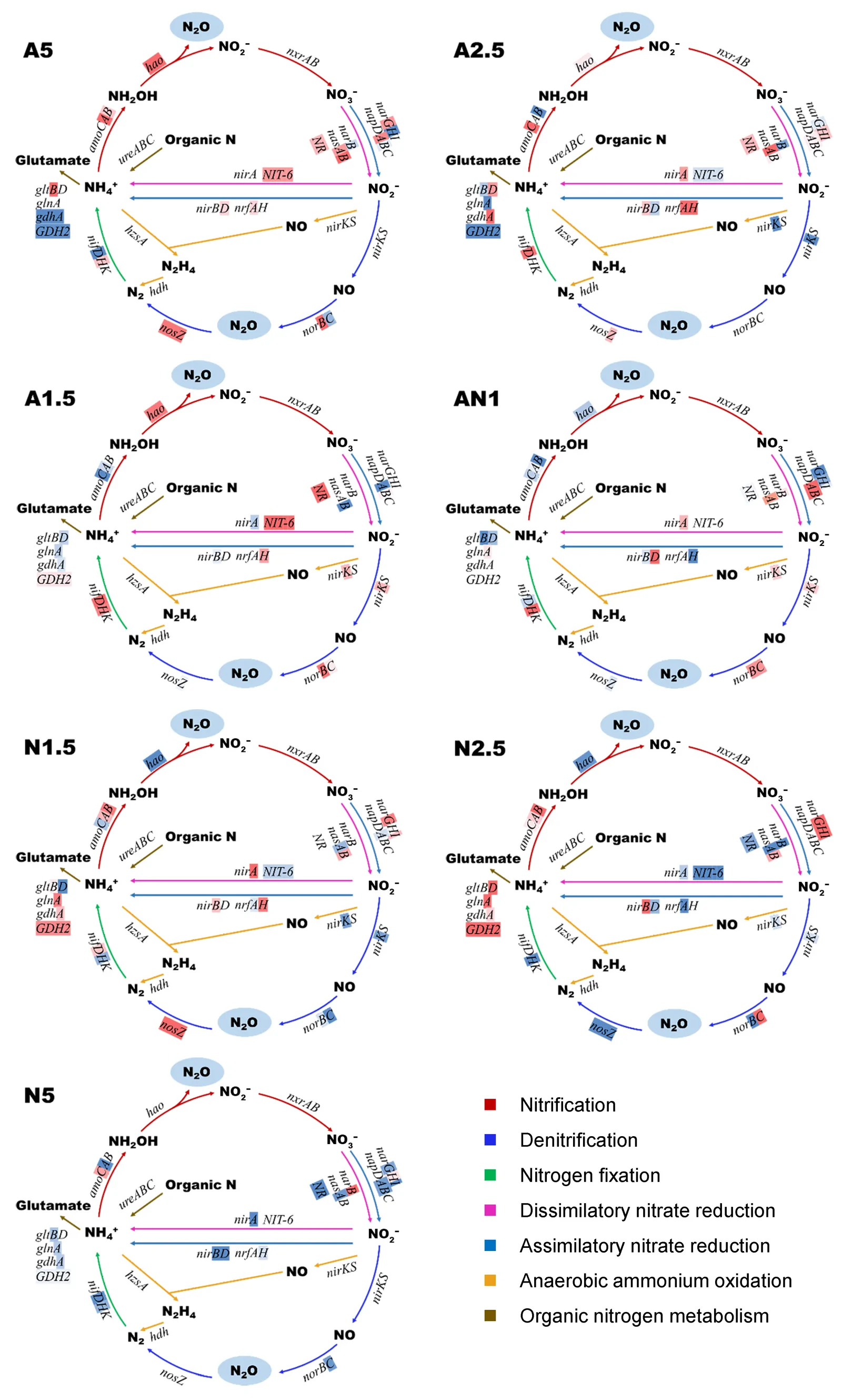
Responses of soil N-transformation genes to different NH_4_^+^/NO_3_^−^ input ratios. CK is the untreated control; A and N indicate NH_4_^+^-N and NO_3_^−^-N additions, respectively, and treatment numbers denote NH_4_^+^/NO_3_^−^ ratios. Changes in functional genes are expressed as (treatment-control)/control. Red indicates lower abundance than CK and blue indicates higher abundance; darker shading corresponds to a larger magnitude of change. Cells without background shading indicate no significant difference from CK. Arrows of different colors indicate different N transformation processes.

Genes central to nitrification included those encoding ammonia monooxygenase (*amoA*, *amoB*, and *amoC*), hydroxylamine oxidoreductase (*hao*), and nitrite oxidoreductase (*nxrAB*). As the NH_4_^+^/NO_3_^−^ ratio declined, the abundance of nitrification-related genes first increased and then decreased. Their greatest abundance occurred at ratios near 1, with particularly clear increases in A1.5, AN1, and N1.5. By contrast, treatments A5 and N2.5, characterized here as having high NH_4_^+^ inputs, showed lower nitrification potential (Figure 6). The pattern indicates that nitrification potential varied with NH_4_^+^ supply and that both very high and very low NH_4_^+^ inputs were unfavorable for nitrifying microorganisms.

The denitrification gene set included nitrate reductases (*narG* and *napA*), nitrite reductase (*nirK*), and nitric oxide reductase (*norB*). Relative abundances of these genes were lower in treatments with NH_4_^+^/NO_3_^−^ ratios > 1 and higher when the ratio was <1. In N5, several denitrification genes, including *narG*, *napA*, *norB*, and *nosZ*, increased significantly, consistent with greater denitrification potential at lower NH_4_^+^/NO_3_^−^ ratios (Figure 6).

Biological N fixation is mediated by the nitrogenase complex, for which *nifD*, *nifK*, and *nifH* are key genes. The relative abundance of N-fixation genes was generally suppressed when NH_4_^+^/NO_3_^−^ ratios were >1 and increased when the ratios were <1 (Figure 6). In particular, *nifH* was significantly more abundant in N1.5, N2.5, and N5 than in the other treatments (Figure 6), suggesting that lower N ratios were more favorable to N-fixing microorganisms.

Although both assimilatory and dissimilatory nitrate reduction convert NO_3_^−^ to NH_4_^+^, they involve distinct gene families and metabolic routes. In general, nitrate-reduction genes were less abundant at NH_4_^+^/NO_3_^−^ ratios > 1 and more abundant at ratios < 1. Under N5, all assayed assimilatory and dissimilatory genes except *narB* increased significantly (Figure 6), demonstrating a strong response of nitrate-reduction potential to N-source composition.

Genes involved in organic N metabolism also varied among treatments. Glutamate synthase (*gltBD*) and glutamine synthase (*glnA*) were more abundant in A5 and A2.5, whereas several organic-N-metabolism genes were suppressed in N1.5 and N2.5 (Figure 6). Smaller increases in some of these genes were observed in A1.5, AN1, and N5 (Figure 6). Together, these patterns indicate that the relative supply of inorganic N forms influenced the abundance of genes involved in organic N metabolism.

## 4. Discussion

### 4.1. Responses of Soil Microbial Communities to the NH_4_^+^/NO_3_^−^ Input Balance

Changing the NH_4_^+^/NO_3_^−^ supply altered the taxonomic organization of the forest-soil microbiome, with especially clear responses among archaeal and eukaryotic groups (Figure 2). This pattern supports the compositional aspect of our first hypothesis. In contrast, α-diversity did not differ detectably among treatments (Table 3), indicating that taxonomic restructuring occurred without a corresponding change in the measured within-sample diversity. Redistribution of taxa could change how N-cycling functions are represented within the community, but unchanged α-diversity by itself does not demonstrate functional redundancy [36].

A decline in the NH_4_^+^/NO_3_^−^ input ratio was accompanied by a relative increase in Thaumarchaeota and decreases in Euryarchaeota and Crenarchaeota. The pattern indicates that archaeal lineages responded differently to inorganic-N composition, but it cannot by itself be taken as proof of substrate preference or competitive superiority. Previous work has associated NH_4_^+^ availability with AOA and AOB, whereas nitrate supply can influence denitrifiers and microorganisms capable of DNRA [37,38,39,40]. The strength and even direction of these responses can depend on the taxa present and on local soil conditions. Studies of ammonia oxidizers and nitrate-responsive microorganisms likewise show that microbial groups do not necessarily respond uniformly to a given N form [24,39].

Several mechanisms, none mutually exclusive, may underlie the observed community reorganization. First, changing the relative supply of NH_4_^+^ and NO_3_^−^ modifies the substrate environment for ammonia oxidation, denitrification, and DNRA [40]. Second, N addition can alter soil acidity and mineral-N availability and thereby affect community composition indirectly [41,42]. Such effects should be interpreted in relation to the measured soil properties rather than assuming a fixed pH response to either N form. Third, differences among archaeal groups and between fungal lineages such as Ascomycota and Basidiomycota may arise from contrasting ecological requirements and strategies for acquiring N [43]. Overall, our data link N-input composition with microbial community restructuring and with changes in inferred N-cycling potential, but they do not directly demonstrate discrete microbial niches or functional redundancy.

Because the experiment applied only one total N loading (80 kg N ha^−1^ yr^−1^) and lasted 84 days under laboratory conditions, the results represent short-term responses to a defined treatment rather than long-term trajectories of atmospheric deposition. Experiments spanning several total-N levels, together with field observations, are needed to determine whether the observed community patterns persist across seasons and environmental contexts. Longer-term measurements would also be required to establish whether these compositional shifts translate into sustained changes in microbial functioning.

### 4.2. Elevated N_2_O Emissions at an NH_4_^+^/NO_3_^−^ Ratio of 1:1 and Their Association with Nitrification Potential

Among all treatments, the 1:1 NH_4_^+^/NO_3_^−^ input produced the largest N_2_O emissions and was accompanied by greater abundances of genes involved in ammonia oxidation, including *amoA*, *amoB*, *amoC*, and *hao* (Figure 1). This enrichment is compatible with higher nitrification potential; however, metagenomic gene abundance does not measure nitrification rate or establish how much nitrification contributed to the observed N_2_O. Denitrification could also have contributed to the flux. Moreover, the maximum at an intermediate input ratio contrasts with the monotonic rise in N_2_O expected under our second hypothesis as nitrate became increasingly dominant.

A possible explanation is that N_2_O formation responded to the simultaneous availability of substrates for ammonia oxidation and denitrification. Under the 1:1 treatment, both inorganic N forms were supplied at equal proportions, potentially supporting concurrent microbial transformations that can generate N_2_O. Previous studies have reported nonlinear N_2_O responses to N availability and have considered substrate balance as a control on nitrification and denitrification [44,45,46,47]. Those findings provide useful context, but they do not prove that the two pathways were coupled in the present incubation. NH_4_^+^ is a substrate for ammonia oxidation, whereas NO_3_^−^ can function as an electron acceptor in denitrification [48,49]. The higher abundance of amo-related genes and *hao* at 1:1 is therefore consistent with greater genetic capacity for ammonia oxidation [50]. Additional possibilities include substrate limitation when NH_4_^+^ is scarce and inhibition at higher concentrations [51], although neither a maximum process rate nor osmotic stress was measured in this study.

**Figure 7:**
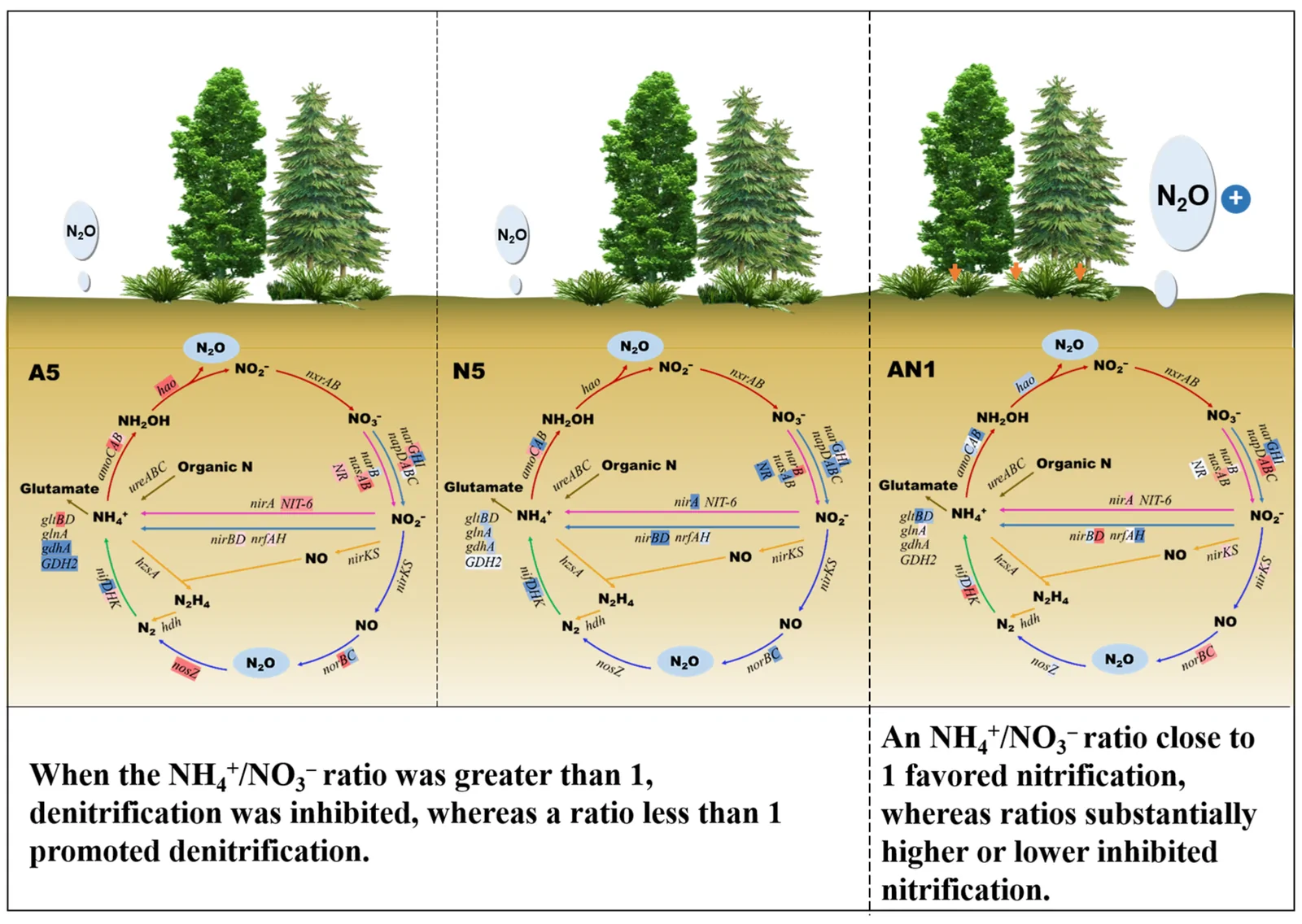
Conceptual representation of proposed relationships among NH_4_^+^/NO_3_^−^ input ratios, microbial N-transformation pathways, and N_2_O emissions in temperate forest soil. Contributions of individual pathways are inferred and were not measured directly. Arrows of different colors indicate different nitrogen transformation processes.

Because regional observations indicate a continuing decline in atmospheric NH_4_^+^/NO_3_^−^ ratios, the response observed at an intermediate ratio is relevant when considering future changes in N deposition [4]. Even so, the 1:1 emission maximum measured in the laboratory should not be interpreted to mean that forest soils necessarily become N_2_O hotspots whenever atmospheric inputs approach this composition. Accordingly, Figure 7 presents hypothesized links among N supply, microbial pathways, and N_2_O release rather than directly measured pathway-specific fluxes.

The experimental soil was air-dried before incubation, but it was subsequently rewetted and held in the dark for a 7-day pre-incubation before N treatments and N_2_O measurements began. This acclimation step was intended to lessen the immediate microbial and mineralization pulse caused by rewetting. The early N_2_O maximum recorded during the formal experiment therefore occurred after the dry–rewet transition rather than immediately at rewetting. Nevertheless, drying and rewetting can leave persistent effects on microbial composition and substrate availability, and a one-week recovery period may not fully return the soil to its original field-moist state. A legacy effect of pretreatment therefore cannot be excluded, and the magnitude and timing of the laboratory N_2_O response should be extrapolated cautiously to intact forest soils receiving natural atmospheric N deposition.

The incubation system also omitted several processes that operate in the field, including plant N uptake, seasonal temperature dynamics, and repeated wetting–drying events. Each of these factors can alter substrate supply and gaseous N losses. Field observations conducted across N-loading gradients and seasons, preferably in combination with assays that distinguish individual pathways, will be necessary to determine whether the response observed at 1:1 is reproduced under natural conditions.

Despite the clear response of N_2_O emissions to the NH_4_^+^/NO_3_^−^ ratio in this experiment, important uncertainties remain. Controlled laboratory incubation cannot reproduce the full set of interactions among soil microorganisms, plants, and climatic variability that occur in natural forests, including root N uptake and wetting–drying cycles. These field processes may further modify nitrification and N_2_O fluxes. Verification will therefore require long-term field experiments and ecological models that represent more complex environmental interactions.

### 4.3. Lower N_2_O Emissions at an NH_4_^+^/NO_3_^−^ Ratio of 1:2.5 and Greater N_2_O-Reduction Potential

The 1:2.5 treatment generated comparatively low N_2_O emissions and simultaneously showed greater abundance of *nosZ*, which encodes the reduction of N_2_O to N_2_. This association is consistent with the possibility that a larger microbial capacity for N_2_O consumption contributed to the lower net flux. However, it does not demonstrate that complete denitrification increased or that the rate of N_2_O-to-N_2_ conversion was directly enhanced. The interpretation is nevertheless consistent with previous work emphasizing the importance of N_2_O-reducing microorganisms [52,53,54].

The effect of nitrate supply on N_2_O reduction may further depend on electron-donor availability, oxygen status, and the composition of the denitrifying community. Studies in other soils have examined how NO_3_^−^ availability regulates denitrification and N_2_O reduction and have also identified circumstances in which additional nitrate alters or constrains microbial activity [55,56,57,58]. In our experiment, the difference between the 1:2.5 and 1:5 treatments shows that increasing the nitrate fraction did not lead to a uniform N_2_O response. The enrichment of DNRA-related genes at 1:5 provides additional evidence that potential nitrate-processing routes shifted across treatments, although it does not establish that DNRA itself generated the measured N_2_O.

At least three related mechanisms could be involved. First, greater *nosZ* abundance may reflect a larger pool of organisms with the genetic capacity to consume N_2_O, thereby lowering net emissions [56]. Second, competition among nitrate-reducing pathways may change with the relative availability of substrates and electron acceptors, although neither *nosZ* expression nor electron transfer was quantified here [57,58]. Third, treatments may differ in the composition of organisms capable of complete versus incomplete denitrification [59]. The available data do not demonstrate selective inhibition of fungal denitrifiers or directly measured enhancement of the N_2_O-reduction rate in the 1:2.5 treatment.

Because all amended treatments received the same total amount of N, differences in N_2_O release indicate that N form may influence emissions independently of N quantity. Whether manipulating N formulations can reduce field emissions from forest or agricultural systems remains uncertain; evidence from this incubation alone is insufficient to establish an effective management practice (Figure 7). Future work should combine measurements of N_2_O and N_2_ fluxes with isotope tracing, gene-expression analyses, and direct process-rate assays so that nitrification-derived N_2_O can be separated from denitrification and N_2_O consumption. DNRA-related gene abundance should be interpreted as functional potential rather than a measured contribution to gas formation. Similarly, the absence of detectable anammox-associated genes does not rule out low-abundance organisms or activity below the detection threshold. Direct pathway-specific evidence will be required before quantitative roles can be assigned to the processes proposed here.

## 5. Conclusions

We combined controlled soil incubation with metagenomic profiling to determine how changes in the NH_4_^+^/NO_3_^−^ input balance influence N_2_O emissions and microbial communities in a temperate forest soil. Altering the relative supply of the two inorganic N forms changed microbial community composition without producing a significant shift in α-diversity. The largest N_2_O release occurred when NH_4_^+^ and NO_3_^−^ were supplied equally (1:1), a pattern that may be associated with nitrification-related processes. At an input ratio of 1:2.5, N_2_O emissions were lower and genes linked to N_2_O reduction were more abundant, consistent with greater microbial potential for N_2_O consumption. More nitrate-rich input was accompanied by enrichment of DNRA-associated genes, indicating altered potential for this pathway without demonstrating a direct contribution of DNRA to N_2_O production. Taken together, the results show that inorganic-N composition is an important factor to consider when assessing microbial N cycling and N_2_O dynamics under changing deposition regimes. Field validation and direct measurements of microbial transformation rates are needed before these findings can be translated into recommendations for N-input management or emission mitigation.

## Figures and Tables

**Table 1:** Soil physicochemical characteristics after incubation under contrasting NH_4_^+^/NO_3_^−^ input ratios.

NH_4_^+^/NO_3_^−^	pH	NO_3_^−^-N (mg kg^−1^)	NH_4_^+^-N (mg kg^−1^)	DOC (mg kg^−1^)	TC (g kg^−1^)	TN (g kg^−1^)	C/N Ratio
CK	4.81 ± 0.03 a	20.75 ± 0.92 a	46.06 ± 1.98 b	12.77 ± 0.73 a	85.33 ± 2.98 a	7.94 ± 0.05 a	10.75 ± 0.31 a
A5	4.82 ± 0.02 a	19.34 ± 0.69 a	52.40 ± 0.89 a	12.94 ± 0.13 a	88.42 ± 0.39 a	7.91 ± 0.07 a	11.18 ± 0.13 a
A2.5	4.82 ± 0.01 a	19.71 ± 0.13 a	51.54 ± 1.18 a	13.06 ± 0.11 a	86.84 ± 2.36 a	7.80 ± 0.03 a	11.13 ± 0.32 a
A1.5	4.80 ± 0.01 a	19.61 ± 0.34 a	52.21 ± 0.52 a	13.12 ± 0.24 a	91.21 ± 0.59 a	7.87 ± 0.10 a	11.59 ± 0.09 a
AN1	4.82 ± 0.01 a	19.82 ± 0.31 a	50.37 ± 0.23 a	12.86 ± 0.42 a	92.84 ± 0.63 a	8.10 ± 0.09 a	11.47 ± 0.21 a
N1.5	4.83 ± 0.03 a	19.64 ± 0.48 a	49.17 ± 0.51 ab	13.40 ± 0.54 a	87.27 ± 0.69 a	7.75 ± 0.09 a	11.27 ± 0.14 a
N2.5	4.84 ± 0.02 a	20.06 ± 1.08 a	51.64 ± 1.70 a	13.46 ± 0.51 a	89.09 ± 0.57 a	7.94 ± 0.09 a	11.23 ± 0.15 a
N5	4.84 ± 0.01 a	17.98 ± 0.39 a	49.30 ± 0.38 ab	13.23 ± 0.28 a	90.81 ± 2.19 a	7.87 ± 0.06 a	11.55 ± 0.36 a
F_7,24_	0.44	1.57	3.76	0.35	2.42	1.85	1.34
*p*	0.86	0.21	0.01	0.92	0.068	0.15	0.30

Note: CK is the untreated control without N addition; A and N denote NH_4_^+^-N and NO_3_^−^-N additions, respectively, and the numbers identify the NH_4_^+^/NO_3_^−^ ratios. Data are means ± SE (*n* = 4). Different lowercase letters indicate significant differences among treatments based on Duncan’s multiple range test (*p* < 0.05).

**Table 2:** Read numbers assigned to major soil microbial domains under different NH_4_^+^/NO_3_^−^ input ratios.

NH_4_^+^/NO_3_^−^ Ratio	Archaea	Bacteria	Eukaryotes
CK	38,647	19,120,583	74,990
A5	33,523	18,524,648	64,140
A2.5	37,356	18,734,504	71,266
A1.5	39,221	16,415,362	64,577
AN1	42,814	19,928,455	77,854
N1.5	33,663	15,537,747	63,717
N2.5	25,290	15,659,467	61,638
N5	36,022	16,129,791	76,547

Note: CK denotes the untreated control; A and N correspond to NH_4_^+^-N and NO_3_^−^-N additions, respectively, and the treatment numbers give the NH_4_^+^/NO_3_^−^ ratios.

**Table 3:** α-Diversity indices for archaea, bacteria, and eukaryotes under contrasting NH_4_^+^/NO_3_^−^ ratios.

NH_4_^+^/NO_3_^−^ Ratio	Archaea		Bacteria		Eukaryotes	
	Shannon	Sobs	Shannon	Sobs	Shannon	Sobs
CK	3.07 ± 0.16 a	830.00 ± 11.06 a	6.63 ± 0.05 a	22,099.00 ± 70.44 a	5.02 ± 0.12 a	1342.00 ± 7.23 a
A5	3.18 ± 0.15 a	829.00± 18.45 a	6.66 ± 0.01 a	22,042.33 ± 239.14 a	5.01 ± 0.09 a	1280.00 ± 21.07 a
A2.5	2.85 ± 0.09 a	809.00 ± 16.50 a	6.63 ± 0.01 a	21,913.33 ± 182.46 a	4.96± 0.03 a	1291.00 ± 21.08 a
A1.5	2.78 ± 0.12 a	813.33 ± 17.49 a	6.65 ± 0.00 a	21,816.00 ± 80.65 a	5.04 ± 0.04 a	1314.33 ± 39.18 a
AN1	2.96 ± 0.08 a	842.67 ± 28.17 a	6.67± 0.02 a	22,193.33± 109.85 a	5.00± 0.05 a	1322.00 ± 9.02 a
N1.5	2.94 ± 0.21 a	776.33 ± 29.16 a	6.58 ± 0.05 a	21,599.00 ± 137.85 a	5.07 ± 0.07 a	1271.33 ± 36.02 a
N2.5	3.25 ± 0.18 a	771.33 ± 12.7 a	6.51 ± 0.09 a	21,627.33 ± 78.88 a	4.96 ± 0.05 a	1230.00 ± 31.09 a
N5	2.82 ± 0.15 a	801.00 ± 6.81 a	6.68 ± 0.02 a	21,755.33 ± 166.26 a	4.97 ± 0.08 a	1298.00 ± 10.60 a
F_7, 24_	1.33	1.80	1.80	2.30	0.30	1.93
*p*	0.28	0.14	0.13	0.06	0.95	0.11

Note: CK is the untreated control without N addition; A and N represent NH_4_^+^-N and NO_3_^−^-N additions, respectively, and treatment numbers indicate the NH_4_^+^/NO_3_^−^ ratios. Sobs is the total number of detected species. Values are means ± standard error. Different lowercase letters denote significant differences among treatments according to Duncan’s multiple range test (*p* < 0.05).

## Data Availability

The datasets generated and analyzed during the current study are available from the corresponding author upon reasonable request, subject to the data management requirements of the supporting research project.

## Supplementary Materials

The following supporting information can be downloaded at: https://www.mdpi.com/article/10.3390/microorganisms14102323/s1, Table S1: Functional genes related to N metabolism (KO00910) in the KEGG database.

## References

[1] Rizwan M., Tanveer H., Ali M.H., Sanaullah M., Wakeel A. (2024). Role of reactive nitrogen species in changing climate and future concerns of environmental sustainability. Environ. Sci. Pollut. Res..

[2] Galloway J.N., Townsend A.R., Erisman J.W., Bekunda M., Cai Z., Freney J.R., Martinelli L.A., Seitzinger S.P., Sutton M.A. (2008). Transformation of the nitrogen cycle: Recent trends, questions, and potential solutions. Science.

[3] Liu X., Zhang Y., Han W., Tang A., Shen J., Cui Z., Vitousek P., Erisman J.W., Goulding K., Christie P. (2013). Enhanced nitrogen deposition over China. Nature.

[4] Yu G., Jia Y., He N., Zhu J., Chen Z., Wang Q., Piao S., Liu X., He H., Guo X. (2019). Stabilization of atmospheric nitrogen deposition in China over the past decade. Nat. Geosci..

[5] Myneni R.B., Dong J., Tucker C.J., Kaufmann R.K., Kauppi P.E., Liski J., Zhou L., Alexeyev V., Hughes M.K. (2001). A large carbon sink in the woody biomass of Northern forests. Proc. Natl. Acad. Sci. USA.

[6] Lu X., Vitousek P.M., Mao Q., Gilliam F.S., Luo Y., Turner B.L., Zhou G., Mo J. (2021). Nitrogen deposition accelerates soil carbon sequestration in tropical forests. Proc. Natl. Acad. Sci. USA.

[7] Yan G., Luo X., Liang C., Han S., Liu G., Yin L., Wang X., Zhang Z., Xu L., Xing Y. (2025). Nitrogen deposition enhances soil organic carbon sequestration through plant–soil–microbe synergies. J. Ecol..

[8] Liu L., Greaver T.L. (2009). A review of nitrogen enrichment effects on three biogenic GHGs: The CO_2_ sink may be largely offset by stimulated N_2_O and CH_4_ emission. Ecol. Lett..

[9] De Vries W., Du E., Butterbach-Bahl K. (2014). Short and long-term impacts of nitrogen deposition on carbon sequestration by forest ecosystems. Curr. Opin. Environ. Sustain..

[10] Lu M., Yang Y., Luo Y., Fang C., Zhou X., Chen J., Yang X., Li B. (2011). Responses of ecosystem nitrogen cycle to nitrogen addition: A meta-analysis. New Phytol..

[11] Liu S., Lin F., Wu S., Ji C., Sun Y.I., Jin Y., Li S., Li Z., Zou J. (2017). A meta-analysis of fertilizer-induced soil NO and combined NO+N_2_O emissions. Glob. Change Biol..

[12] You L., Ros G.H., Chen Y., Yang X., Cui Z., Liu X., Jiang R., Zhang F., De Vries W. (2022). Global meta-analysis of terrestrial nitrous oxide emissions and associated functional genes under nitrogen addition. Soil Biol. Biochem..

[13] Gao W., Kou L., Zhang J., Müller C., Wang H., Yang H., Li S. (2016). Enhanced deposition of nitrate alters microbial cycling of N in a subtropical forest soil. Biol. Fertil. Soils.

[14] Geng J., Cheng S., Fang H., Yu G., Li X., Si G., He S., Yu G. (2017). Soil nitrate accumulation explains the nonlinear responses of soil CO_2_ and CH_4_ fluxes to nitrogen addition in a temperate needle-broadleaved mixed forest. Ecol. Indic..

[15] Nie Y., Wang M., Zhang W., Ni Z., Hashidoko Y., Shen W. (2018). Ammonium nitrogen content is a dominant predictor of bacterial community composition in an acidic forest soil with exogenous nitrogen enrichment. Sci. Total Environ..

[16] Lu M., Cheng S., Fang H., Yang Y., Guo Y., Li Y., Zhou Y. (2022). Contrasting response of soil N_2_O release to ammonium, nitrate, and urea addition rates is determined by substrate availability and microbial community abundance and composition. Eur. J. Soil Biol..

[17] Guo Y., Geng J., Cheng S., Fang H., Li X., Yang Y., Li Y., Zhou Y. (2023). Soil acidification and ammonia-oxidizing archaeal abundance dominate the contrasting responses of soil N_2_O emissions to NH_4_^+^ and NO_3_^−^ enrichment in a subtropical plantation. Eur. J. Soil Biol..

[18] Frey S.D., Knorr M., Parrent J.L., Simpson R.T. (2004). Chronic nitrogen enrichment affects the structure and function of the soil microbial community in temperate hardwood and pine forests. For. Ecol. Manag..

[19] Lu M., Cheng S., Fang H., Xu M., Yang Y., Li Y., Zhang J., Müller C. (2021). Organic nitrogen addition causes decoupling of microbial nitrogen cycles by stimulating gross nitrogen transformation in a temperate forest soil. Geoderma.

[20] Liu W., Jiang L., Yang S., Wang Z., Tian R., Peng Z., Chen Y., Zhang X., Kuang J., Ling N. (2020). Critical transition of soil bacterial diversity and composition triggered by nitrogen enrichment. Ecology.

[21] Ma X., Wang T., Shi Z., Chiariello N.R., Docherty K., Field C.B., Gutknecht J., Gao Q., Gu Y., Guo X. (2022). Long-term nitrogen deposition enhances microbial capacities in soil carbon stabilization but reduces network complexity. Microbiome.

[22] Chen W., Hou Z., Zhang D., Wang K., Xing J., Song Y. (2025). The complex co-occurrence network under N deposition resulting in the change of soil bacterial structure and the decrease of bacterial abundance in subtropical *Quercus aquifolioides* forest. Forests.

[23] Shi X., Wang L., Sun J., Lucas-Borja M.E., Wang J. (2023). Nitrogen cycling-related functional genes exhibit higher sensibility in soil than leaf phyllosphere of different tree species in the subtropical forests. Plant Soil.

[24] Frey B., Varliero G., Qi W., Stierli B., Walthert L., Brunner I. (2022). Shotgun metagenomics of deep forest soil layers show evidence of altered microbial genetic potential for biogeochemical cycling. Front. Microbiol..

[25] Fierer N., Lauber C.L., Ramirez K.S., Zaneveld J., Bradford M.A., Knight R. (2012). Comparative metagenomic, phylogenetic and physiological analyses of soil microbial communities across nitrogen gradients. ISME J..

[26] Li B.B., Roley S.S., Duncan D.S., Guo J., Quensen J.F., Yu H.Q., Tiedje J.M. (2021). Long-term excess nitrogen fertilizer increases sensitivity of soil microbial community to seasonal change revealed by ecological network and metagenome analyses. Soil Biol. Biochem..

[27] Hu H.W., Chen D., He J.Z. (2015). Microbial regulation of terrestrial nitrous oxide formation: Understanding the biological pathways for prediction of emission rates. FEMS Microbiol. Rev..

[28] Barnard R., Leadley P.W., Hungate B.A. (2005). Global change, nitrification, and denitrification: A review. Glob. Biogeochem. Cycles.

[29] Brochier-Armanet C., Boussau B., Gribaldo S., Forterre P. (2008). Mesophilic Crenarchaeota: Proposal for a third archaeal phylum, the Thaumarchaeota. Nat. Rev. Microbiol..

[30] Schreiber F., Wunderlin P., Udert K.M., Wells G.F. (2012). Nitric oxide and nitrous oxide turnover in natural and engineered microbial communities: Biological pathways, chemical reactions, and novel technologies. Front. Microbiol..

[31] Baggs E.M. (2011). Soil microbial sources of nitrous oxide: Recent advances in knowledge, emerging challenges and future direction. Curr. Opin. Environ. Sustain..

[32] Zhang L.M., Hu H.W., Shen J.P., He J.Z. (2012). Ammonia-oxidizing archaea have more important role than ammonia-oxidizing bacteria in ammonia oxidation of strongly acidic soils. ISME J..

[33] Guo Y., Cheng S., Fang H., Yang Y., Li Y., Shi F., Zhou Y. (2023). Copper and cadmium co-contamination affects soil bacterial taxonomic and functional attributes in paddy soils. Environ. Pollut..

[34] Guo Y., Cheng S., Fang H., Geng J., Li Y., Shi F., Wang H., Chen L., Zhou Y. (2024). Copper and cadmium co-contamination increases the risk of nitrogen loss in red paddy soils. J. Hazard. Mater..

[35] R Core Team (2020). R: A Language and Environment for Statistical Computing.

[36] Yu L., Luo S., Gou Y., Xu X., Wang J. (2021). Structure of rhizospheric microbial community and N cycling functional gene shifts with reduced N input in sugarcane-soybean intercropping in South China. Agric. Ecosyst. Environ..

[37] Ouyang Y., Norton J.M., Stark J.M., Reeve J.R., Habteselassie M.Y. (2016). Ammonia-oxidizing bacteria are more responsive than archaea to nitrogen source in an agricultural soil. Soil Biol. Biochem..

[38] Liao Y., He T., Wang C., Zheng C., Zhang M. (2025). Dissimilatory nitrate reduction to ammonium has a competitive advantage over denitrification under nitrate-limited conditions. Rev. Environ. Sci. Bio/Technol..

[39] Bates S.T., Berg-Lyons D., Caporaso J.G., Walters W.A., Knight R., Fierer N. (2011). Examining the global distribution of dominant archaeal populations in soil. ISME J..

[40] Putz M., Schleusner P., Rütting T., Hallin S. (2018). Relative abundance of denitrifying and DNRA bacteria and their activity determine nitrogen retention or loss in agricultural soil. Soil Biol. Biochem..

[41] Guo P., Yang L., Kong D., Zhao H. (2022). Differential effects of ammonium and nitrate addition on soil microbial biomass, enzymatic activities, and organic carbon in a temperate forest in North China. Plant Soil.

[42] Zhang L., Zhao Z., Jiang B., Baoyin B., Cui Z., Wang H., Li Q., Cui J. (2024). Effects of long-term application of nitrogen fertilizer on soil acidification and biological properties in China: A meta-analysis. Microorganisms.

[43] He J., Jiao S., Tan X., Wei H., Ma X., Nie Y., Liu J., Lu X., Mo J., Shen W. (2021). Adaptation of soil fungal community structure and assembly to long-versus short-term nitrogen addition in a tropical forest. Front. Microbiol..

[44] McSwiney C.P., Robertson G.P. (2005). Nonlinear response of N_2_O flux to incremental fertilizer addition in a continuous maize (*Zea mays* L.) cropping system. Glob. Change Biol..

[45] Tian X.F., Hu H.W., Ding Q., Song M.H., Xu X.L., Zheng Y., Guo L.D. (2014). Influence of nitrogen fertilization on soil ammonia oxidizer and denitrifier abundance, microbial biomass, and enzyme activities in an alpine meadow. Biol. Fertil. Soils.

[46] Carey C.J., Dove N.C., Beman J.M., Hart S.C., Aronson E.L. (2016). Meta-analysis reveals ammonia-oxidizing bacteria respond more strongly to nitrogen addition than ammonia-oxidizing archaea. Soil Biol. Biochem..

[47] Sun Y., Sheng S., Jiang X., Bello A., Wu X., Meng Q., Deng L., Xu X., Li H. (2019). Genetic associations as indices for assessing nitrogen transformation processes in co-composting of cattle manure and rice straw. Bioresour. Technol..

[48] Zhang Y., Guo G., Wu H., Mu Y., Liu P., Liu J., Zhang C. (2019). The coupling interaction of NO_2_^−^ with NH_4_^+^ or NO_3_^−^ as an important source of N_2_O emission from agricultural soil in the North China Plain. Sci. Total Environ..

[49] Zhang Y., Song C., Zhou Z., Cao X., Zhou Y. (2019). Coupling between nitrification and denitrification as well as its effect on phosphorus release in sediments of Chinese Shallow Lakes. Water.

[50] Song L., Niu S. (2022). Increased soil microbial AOB amoA and narG abundances sustain long-term positive responses of nitrification and denitrification to N deposition. Soil Biol. Biochem..

[51] Norton J., Ouyang Y. (2019). Controls and adaptive management of nitrification in agricultural soils. Front. Microbiol..

[52] Philippot L., Čuhel J., Saby N.P., Chèneby D., Chroňáková A., Bru D., Arrouays D., Martin-Laurent F., Šimek M. (2009). Mapping field-scale spatial patterns of size and activity of the denitrifier community. Environ. Microbiol..

[53] Zhang B., Zhou M., Zhu B., Kemmann B., Pfülb L., Burkart S., Liu H.B., Butterbach-Bahl K., Well R. (2023). Threshold-like effect of soil NO_3_^−^ concentrations on denitrification product N_2_O/(N_2_O + N_2_) ratio is mediated by soil pH. Soil Biol. Biochem..

[54] Li Z., Yang Y., Wang X., Qi Y., Yang X. (2025). Linking N_2_O emissions and nosZ gene abundance: A meta-analysis of organic carbon amendments in agricultural soils. Plant Soil.

[55] Wallenstein M.D., Myrold D.D., Firestone M., Voytek M. (2006). Environmental controls on denitrifying communities and denitrification rates: Insights from molecular methods. Ecol. Appl..

[56] Chen Z., Wang C., Gschwendtner S., Willibald G., Unteregelsbacher S., Lu H., Kolar A., Schloter M., Butterbach-Bahl K., Dannenmann M. (2015). Relationships between denitrification gene expression, dissimilatory nitrate reduction to ammonium and nitrous oxide and dinitrogen production in montane grassland soils. Soil Biol. Biochem..

[57] Shan J., Sanford R.A., Chee-Sanford J., Ooi S.K., Löffler F.E., Konstantinidis K.T., Yang W.H. (2021). Beyond denitrification: The role of microbial diversity in controlling nitrous oxide reduction and soil nitrous oxide emissions. Glob. Change Biol..

[58] Zhao S., Liu Y., Xu L., Ye J., Zhang X., Xu X., Meng H., Xie W., He H., Wang G. (2024). NosZ II/nosZ I ratio regulates the N_2_O reduction rates in the eutrophic lake sediments. Sci. Total Environ..

[59] Wan Z., Wang L., Huang G., Rasul F., Awan M.I., Cui H., Liu K., Yu X., Tang H., Wang S. (2023). nirS and nosZII bacterial denitrifiers as well as fungal denitrifiers are coupled with N_2_O emissions in long-term fertilized soils. Sci. Total Environ..

