# Supplementary material for: Short-Term Simulated NH4+/NO3− Inputs Reorganize Soil Microbial Communities and Modify N-Cycling Functional Potential in Temperate Forests
Source: Microorganisms. 2026 Oct 12;14(10):2323. doi: 10.3390/microorganisms14102323 (PMC13707966; doi:10.3390/microorganisms14102323)
Supplement: Supplementary file 1 [file microorganisms-14-02323-s001.zip › microorganisms-4599301-supplementary.pdf]

microorganisms

Table S1. Functional genes related to N metabolism (KO00910) in the KEGG database.

Functional gene (subunit)	Annotation	KEGG number
**Nitrate assimilation salt reduced to ammonium (ANRA)**		
narB	ferredoxin-nitrate reductase	K00367
NR	nitrate reductase (NAD(P)H)	K10534
nasA	assimilatory nitrate reductase catalytic subunit	K00372
nasB	assimilatory nitrate reductase electron transfer subunit	K00360
NIT-6	nitrite reductase (NAD(P)H)	K17877
nirA	ferredoxin-nitrite reductase	K00366
**Dissimilatory nitrate reduction to ammonium (DNRA)**		
nirB	nitrite reductase (NADH) large subunit	K00362
nirD	nitrite reductase (NADH) small subunit	K00363
nrfA	nitrite reductase (cytochrome c-552)	K03385
nrfH	cytochrome c nitrite reductase small subunit	K15876
**Denitrification**		
narG	nitrate reductase / nitrite oxidoreductase, alpha subunit	K00370
narH	nitrate reductase / nitrite oxidoreductase, beta subunit	K00371
narI	nitrate reductase gamma subunit	K00374
napA	periplasmic nitrate reductase NapA	K02567
napB	cytochrome c-type protein NapB	K02568
nirK	nitrite reductase (NO-forming)	K00368
nirS	nitrite reductase (NO-forming) / hydroxylamine reductase	K15864
norB	nitric oxide reductase subunit B	K04561
norC	nitric oxide reductase subunit C	K02305
nosZ	nitrous-oxide reductase	K00376
**N fixation**		
nifD	Nase molybdenum-iron protein alpha chain	K02586
nifK	Nase molybdenum-iron protein beta chain	K02591
nifH	Nase iron protein NifH	K02588
anfG	Nase delta subunit	K00531
**Nitrification**		
amoA	methane/ammonia monooxygenase subunit A	K10944
amoB	methane/ammonia monooxygenase subunit B	K10945
amoC	methane/ammonia monooxygenase subunit C	K10946
hao	hydroxylamine dehydrogenase	K10535
**Anaerobic ammonia oxidation**		
hdh	hydrazine dehydrogenase	K20935
**Organic N transformation**		
gltB	glutamate synthase (NADPH/NADH) large chain	K00265
gltD	(NADPH/NADH) small chain	K00266
GLT1	glutamate synthase (NADPH/NADH)	K00264
E1.4.7.1	glutamate synthase (ferredoxin)	K00284
glnA	glutamine synthetase	K01915
GDH2	glutamate dehydrogenase	K15371
gudB	glutamate dehydrogenase	K00260
gdhA, GLUD1_2	glutamate dehydrogenase (NAD(P)+)	K00261
gdhA, E1.4.1.4	glutamate dehydrogenase (NADP+)	K00262
